# Fast Monte Carlo simulation for total body irradiation using a ${\;}^{60}Co$ teletherapy unit

**DOI:** 10.1120/jacmp.v14i3.4214

**Published:** 2013-05-06

**Authors:** Xiaodong Liu, Danielle Lack, Joseph T. Rakowski, Cory Knill, Michael Snyder

**Affiliations:** ^1^ Department of Radiation Oncology Wayne State University School of Medicine Detroit MI; ^2^ Department of Radiation Oncology Karmanos Cancer Center Detroit MI USA

**Keywords:** Monte Carlo, TBI, cobalt, fast, dose calculation

## Abstract

Our institution delivers TBI using a modified Theratron 780 ${\;}^{60}Co$ unit. Due to limitations of our treatment planning system in calculating dose for this treatment, we have developed a fast Monte Carlo code to calculate dose distributions within the patient. The algorithm is written in C and uses voxel density information from CT images to calculate dose in heterogeneous media. To test the algorithm, film‐based dose measurements were made separately in a simple water phantom with a high‐density insert and a RANDO phantom and then compared to doses calculated by the Monte Carlo algorithm. In addition, a separate simulation in GEANT4 was run for the RANDO phantom and compared to both film and the in‐house simulation. All results were analyzed using RIT113 film analysis software. Simulations in the water phantom accurately predict the depth of maximum dose in the phantom at 0.5 cm. The measured PDD along the central axis of the beam closely matches the PDD generated from the Monte Carlo code, deviating on average by only 3% along the depth of the water phantom. Dose measured at planes inside the high‐density insert had a mean difference of 4.9% on cross‐profile measurement. In the RANDO phantom, gamma pass rates vary between 91% and 99% at 3 mm, 3%, and were >99% at 5 mm, 5% for the four film planes measured. Profiles taken across the film and both simulations resulted in mean relative differences of <2% for all profiles in each slice measured. The Monte Carlo algorithm presented here is potentially a viable method for calculating dose distributions delivered in TBI treatments at our center. While not yet refined enough to be the primary method of treatment planning, the algorithm at its current resolution determines the dose distribution for one patient within a few hours, and provides clinically useful information in planning TBI. With appropriate optimization, the Monte Carlo method presented here could potentially be implemented as a first‐line treatment planning option for ${\;}^{60}Co$ TBI.

PACS number: 87.10.Rt

## I. INTRODUCTION

In a manner similar to other institutions, our center currently offers total body irradiation (TBI) treatment using a Theratron 780 ${\;}^{60}Co$ unit which has been fitted with a custom collimator and treatment table.^(1)^ Treatment planning of our TBI treatments is usually accomplished through simple hand calculations of dose to specific points at depth in the patient. Although these calculations have provided relatively accurate dosimetry for several years, the point dose nature of the calculations provides less than optimal knowledge of the dose distribution within the patient and results in uncertainty in dose to normal tissues — the lungs, in particular.^(2)^ T o reduce or eliminate this uncertainty, we have sought to calculate dose volumetrically as in other conventional external beam treatments.

Our current commercial treatment planning software, Varian's Eclipse 8.9, cannot be used in this application. The calculation algorithms currently available in Eclipse 8.9 are based on beam models derived from data measured at the treatment machine. As Eclipse is designed around Varian's linear accelerators, the calculation algorithms have been optimized for treatment geometries associated with their accelerators. The nonstandard geometry presented by our TBI unit — 50 cm×200 cm field sizes — prevents the use of Eclipse for dose calculation for a typical TBI treatment as delivered at our center. While other, more flexible, treatment planning systems will allow for extended SSD calculations, it is clinically undesirable to maintain multiple commercial treatment planning systems for individual use cases. As such, we have investigated developing an in‐house Monte Carlo‐based solution for TBI dose calculation.^(3)^

Monte Carlo frameworks like GEANT4 and EGS4 have been used successfully in dose calculations at the photon energy in question, however complete treatment planning in this way has been limited by long calculation times.^(4)^ A fast Monte Carlo simulation is desirable to obtain accurate dose calculations on a timescale acceptable in standard clinical practice. To achieve high accuracy in heterogeneous tissue within reasonable clinical time constraints, such an algorithm should be based on voxel density information from CT images of patients, while at the same time employing application specific approximations during the evaluation of particle transport.^5^, ^6^, ^7^ Given the extremely large dose calculation volume for a TBI treatment, a simple algorithm must make a series of reasonable approximations of radiation transport within the volume in order to achieve calculation times that fall within clinically reasonable limits. This certainly, in principle, can be achieved with several existing Monte Carlo codes optimized for voxelized dose calculations (VMC++, XVMC, DPM, etc.).^5^, ^8^, ^9^ However, due to the fact that we wish to model a very specific treatment situation, there are a number of inherent simplifications that can be made that should be best implementable at a low level in the code such that the final dose calculation algorithm is as light as possible.

In this work we have created a simple Monte Carlo algorithm for the purpose of TBI planning on our ${\;}^{60}Co$ unit. We will describe the algorithm and how it has been implemented and the assumptions that have been made. We will then present a comparison of our fast Monte Carlo simulations to: a) measured data for the irradiation of a custom water phantom and an anthropomorphic RANDO phantom, and b) an equivalent simulation performed in GEANT4. Finally, we will discuss the potential of this algorithm and the work that still needs to be completed for the algorithm to be employed in the clinic.

## II. MATERIALS AND METHODS

The overall goal of constructing the Monte Carlo code is to provide a lightweight framework that takes advantage of the specific approximations that can be made in dose calculation due to the clinical situation in question. The pseudo‐monoenergetic source and less restrictive requirements of calculation accuracy present in ${\;}^{60}Co$ TBI treatments should allow for a significant reduction in physics processes and resolution of data tables in the code, while still achieving results more informative than hand calculation of point doses.^(10)^ Below we describe various processes incorporated into the code and the approximations that have been employed.

### A. Beam modeling and physics processes

**Figure 1:**
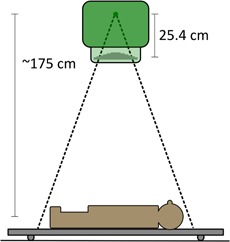
The treatment and simulation setup.

All patients treated on the TBI unit are set up on a custom designed table that sits on the floor of the treatment room. The distance from the ${\;}^{60}Co$ source to the floor is 195.4 cm, and the average distance to patient midplane is ∼175 cm. The patient is set up such that the midpoint of the body aligns with the central axis of the treatment unit. The top of the table on which the patient lies is 6.0 cm from the floor, and the table itself is oriented 90° from the potential axis of rotation of the gantry (the gantry remains vertical during all treatments). A diagram of the treatment setup is presented in Fig. 1.

${\;}^{60}Co$ decays with 0.9988 probability to a particular excited metastable state of ${\;}^{60}Ni$ through beta decay. This excited ${\;}^{60}Ni$ nucleus further decays to its ground state by emitting two gamma rays, each with the same relative abundance differing by only 1 in 1000. The energies of the two gamma rays are 1.17 MeV and 1.33 MeV, respectively. Due to the equal emission of the two gammas, the effective gamma emission of ${\;}^{60}Co$ is often approximated by employing an average gamma energy of 1.25 MeV. For the purposes of simulation, the small gap in energy between the two gammas makes it computationally convenient to use this single energy approximation and average energy of 1.25 MeV.

The custom Monte Carlo code was written in C to simulate energy deposition by 1.25 MeV photons over a broad field defined by our modified collimator on the ${\;}^{60}Co$ unit. The physics processes that can occur during simulation are location‐dependent within the simulation space. In the flattening filter contributions of Rayleigh scattering, photoelectric effect and Compton scattering are included. However, in the patient Rayleigh scattering is excluded due to very low interaction probability in tissue at the initial photon energy. The contribution of pair production is ignored everywhere due to low photon energy.

### B. Construction of source head and filter

The radius of the ${\;}^{60}Co$ source is 2.0 cm. It is located in a lead‐lined chamber thick enough to ignore contributions from head leakage due to gammas not emitted in line with primary collimation. There is a lead flattening filter composed of six round plates with a thickness of 0.3175 cm each. The diameters of the round plates are 7.62, 12.7, 17.8, 22.9, 27.9, and 33.0 cm, respectively. The distance from the source to the bottom of the filter is 25.4 cm. The length and width of the filter is 33.0 cm and 14.0 cm, respectively.

### C. Beam flattening

The simulation begins by randomly selecting an initial momentum for the photon within the limits of collimation. The photon is transported through the lead filter under the approximations that: i) only zero and single scatter photons contribute dose to the patient, and ii) no electrons generated in the filter contribute dose. The second approximation is made due to a thin sheet of acrylic present during treatment that reduces electron contamination in the treatment beam. The effect of this piece of acrylic is assumed to be limited to the electron contamination and overall beam intensity, and is therefore not included explicitly in the simulation.

The probability of collision of an individual gamma with the flattening filter is sampled according to the transmission through the filter:

(1) $$A=e^{‐\mu \cdot t}$$

where μ is the attenuation coefficient of lead and *t* is the thickness of the filter. The physics processes included in the flattening filter transport are the photoelectric effect, Compton scattering, and Rayleigh scattering. Each component is sampled based on its weight in the total attenuation coefficient.^(11)^ The assumption of a single gamma energy allows for the simulation to be simplified to a single lookup table for each process. The contribution of pair production is ignored because of the low photon energy and extremely small contribution to the total attenuation coefficient.

For photoelectric process in the flattening filter, the photon is absorbed and the emitted electron is dismissed. For Compton scattering, the recoil electron is dismissed, and the photon scattering angle is sampled according to the Compton differential cross section

(2) $$\frac{d\sigma }{d\Omega_{c}}=\frac{r_{0}^{2}}{2}{\left(\frac{hv^{\prime }}{hv}\right)}^{2}\left(\frac{hv}{hv^{\prime }}+\frac{hv^{\prime }}{hv}‐{\text{sin}}^{2}\theta \right)$$

where *h*ν is the energy of the incoming photon, ${hv}^{\prime }$ is the energy of the outgoing photon, θ is the angle of deflection, and $r_{0}$ is the classical electron radius given by:

(3) $$r_{0}=\frac{e^{2}}{4\pi \varepsilon_{0}m_{0}c^{2}}=2.8719\times 10^{‐15}m$$

The azimuthal angle Ψ is sampled uniformly from 0° and 360°. The unit vector of the direction of the scattered photon is given by:

(4) $${\hat{u}}^{\prime }(x,y,z)=\Omega (\theta ,\psi )\cdot \hat{u}(x,y,z)$$

where û(x,y,z) is the incoming directional unit vector, and Ω(θ,Ψ) is the rotation matrix given by:

(5) $$\Omega (\theta ,\psi )=\left(\begin{array}{ccc} \text{cos}\theta \text{cos}\psi & \text{cos}\theta \text{sin}\psi & ‐\text{sin}\theta \\ ‐\text{sin}\psi & \text{cos}\psi & 0\\ \text{sin}\theta \text{cos}\psi & \text{sin}\theta \text{sin}\psi & \text{cos}\theta \end{array}\right)$$

The energy of the scattered photon is determined by:

(6) $$hv^{\prime }=\frac{hv}{1+\frac{hv}{m_{0}c^{2}}\left(1‐\text{cos}\theta \right)}$$

For Rayleigh scattering, there is no recoil electron and the photon loses no energy, but a change in the direction of the photon occurs. The scattering angle is sampled according to the Rayleigh differential cross section:^(12)^

(7) $$\frac{\delta \sigma }{d\Omega_{R}}=\left(1‐{\text{cos}}^{2}\theta \right)\cdot \text{sin}\theta \cdot F^{2}\left(q\right)$$

where F(q) is the Hubbell's form factor and *q* is the momentum transfer given by:

(8) $$q=2hv\cdot \text{sin}\left(\frac{\theta }{2}\right)$$

The azimuthal angle is sampled uniformly from 0° and 360°. The new directional unit vector is determined by rotation matrix as described in (4), (5)) above.

All photons through the filter will be projected onto the entry plane of patient. If the incident point is outside the entry window of the patient, the photon is dismissed. This approximation ignores interactions that could take place in air between the patient and the flattening filter.

### D. Voxel‐based Monte Carlo simulation in patient

If a given photon is transported through the flattening filter and remains within the patient window, transport within the patient begins. In order for the Monte Carlo dose algorithm to be clinically useful, it must be able to simulate the transport of photons and electrons based on CT image sets collected during patient treatment simulation. To this end, a separate procedure was created in MATLAB (The MathWorks, Natick, MA) to read CT image sets in DICOM format and create binary data files containing the voxelized information in a format easily inputted into the Monte Carlo algorithm. The translated data file is then read into an array containing an element for each voxel in the original CT dataset. For a given simulation, the patient is represented as lying in a digitized box with the same geometry as the CT image. Each element of the digitized box preserves the Hounsfield's unit (HU) and spatial location of the original CT voxel. To preserve the geometry of treatment, the top of the table on which the patient lies in the CT image set is set 6.0 cm higher than the floor in the simulation to correspond to the table top in the treatment room. For the results presented here, the voxel dimensions were 2.54×2.54×5 mm in X, Y, and Z directions, respectively, where Z is the CT slice thickness.

In order to determine the transport of a given particle, the density and material type must be known. While this is difficult to obtain directly from HU values in a CT image set, a set of empirical assumptions can be used as a good approximation. As in standard treatment planning systems, the density of a given voxel can be related to the HU values given in the CT image set.^(13)^ In the simulation, tabled data for our CT simulator is fit by the bilinear function:

(9) $$p=\{\begin{array}{cc} 0.001\cdot HU+1.034 & HU<=0\\ 0.0006\cdot HU+1.034 & HU>0 \end{array}$$

Once a density is assigned, the type of material can be selected based on that density.^(14)^ The mean free path, Λ, of photon in each voxel is then determined by the mass attenuation coefficients of the material and its density:

(10) $$\frac{1}{\lambda }=\left[{\left(\frac{\mu }{\rho }\right)}_{PE}+{\left(\frac{\mu }{\rho }\right)}_{C}+{\left(\frac{\mu }{\rho }\right)}_{R}\right]\cdot \rho$$

The values of mass attenuation coefficients for different materials are taken from Appendix D2 of the work by Attix.^(11)^

The distance of travel before interaction is determined by accumulating transmission probabilities along the path in tissue:

(11) $$A=\text{exp}\left(‐\sum_{j}^{n}\frac{h}{\lambda_{j}}\right)$$

where the parameter *h* is the step size of particle transportation (chosen as 1 mm for this work), and $\lambda_{j}$ is the mean free path of the photon in the jth voxel. Each photon is assigned a surviving probability by choosing a random number between 0 and 1 using a Mersenne twister pseudo‐random number generator.^(15)^ When the accumulated transmission probability, A, is equal to the randomly chosen surviving probability, a collision for this photon occurs. The length of photon track before the scattering event is the sum of all step sizes before the collision. The process of “stepping” the photon then refers to the iterative, algorithmic process of accumulating transmission probability along the photon track. Due to the possibility of material change along the photon track, a particular sampled surviving probability will not always correspond to the same path length before the next interaction. Here the stepping accounts for this change by allowing the transmission probability to accumulate at each step as a function of the linear attenuation coefficient at each individual step. In this way the particle is stepped along the track until the interaction site is found, and in this sense the step size can be considered the resolution of this process. Despite this iteration, only a single random sampling occurs for each interaction at the beginning of the process when the surviving probability is determined. After an interaction, the scattered photon will be assigned a new surviving probability and the above process is repeated.

If the collision is determined to be a photoelectric process, the photon is completely absorbed and the emitted electron is assigned the same energy as the photon. Due to the low probability of a photoelectric process occurring at the initial 1.25 MeV energy, the majority of photoelectric interactions will take place with scattered photons at much lower energies. As such, these electrons will be ejected with low energies and short ranges. Due to the coarse dose matrix used in our simulation, the energy of these electrons is low enough to confine the electron travel to a single voxel. As an overall approximation in the code, we therefore ignore the angular distribution of the ejected electrons. As a further approximation, the energy loss due to binding energy is ignored.

For Compton scattering, the scattering angle and energy are calculated based on (4), (6). The emitted angle of recoil electron (φ) is determined by:

(12) $$\text{cot}\left(\varphi \right)=\left(1‐\frac{hv}{m_{0}c^{2}}\right)\cdot \text{tan}\left(\frac{\theta }{2}\right)$$

The azimuthal angle of recoil electron is opposite to that of the photon:

(13) $$\psi_{e}=\psi_{ph}+180^{\circ }$$

The direction of recoil electron is determined by rotation matrix in (4), (5). The kinetic energy of the recoil electron is:

(14) $$T_{e}=hv‐hv^{\prime }$$

Due to the low energy of the majority of recoil electrons, transport is carried out using the continuously slowing down approximation (CSDA) without angular scatter. As such, the electron path is followed in a straight line with continuous energy deposition until all kinetic energy is deposited. Using the CSDA approximation without considering angular scatter obviates the computational cost of sampling a multiple‐scattering distribution and significantly speeds up the simulation. The energy deposition from recoil electrons is calculated based on the electron mass collision stopping powers:^(11)^

(15) $$\Delta E={\left(\frac{dT}{\rho dx}\right)}_{c}\cdot h$$

where *h* is the step size (chosen to be 1 mm for this work). The tables of mass collision stopping powers are taken from Appendix E of the work by Attix.^(11)^

The energy cutoff for photon and electron transport is set to 10 keV. When the kinetic energy of a photon or an electron is less than the energy cutoff, the particles are dismissed and their entire energy is deposited in the local voxel. The final dose distribution in voxel (i, j, k) is determined by:

(16) $$D\left(i,j,k\right)=\frac{\Delta E\left(i,j,k\right)}{\Delta m\left(i,j,k\right)}=\frac{\Delta E\left(i,j,k\right)}{V\cdot \rho \left(i,j,k\right)}$$

where ΔE(i,j,k) is the total energy deposited in that voxel, ρ(i,j,k) is the density of that voxel, and *V* is the volume of each voxel. The dose calculated in this way is representative of the dose deposited in the voxel material, but not representative of the way dose is measured and defined in clinic (i.e., dose to water). To convert to dose to water, stopping‐power ratios between water and the voxel material are used as in Bragg‐Gray cavity theory.^16^, ^17^ The dose conversion is done at the end of simulation using stopping power ratios determined at an average energy of 0.3 MeV for aluminum, lung, soft tissue, and cortical bone.

### E. Sampling method

The method used for sampling is essentially a numerical inversion of the discretized cross sections. At a resolution of 1°, each differential cross section is evaluated at a particular angle, summed with all values of the differential cross section between that angle and 0°, and tabled. All tabled values are then normalized to the total cross section. At a sampling resolution of 1000 total bins between [0,1], each angle is then tabled with a frequency in 1000 equal to its probability of occurring as determined through the cross‐sectional tabling. This is repeated for every photon energy, from 0 keV to 1250 keV, at a resolution of 10 keV. In this way, a number sampled from a uniform distribution multiplied by 1000 and rounded to the nearest integer represents an index in the table of outgoing angles. No comparison is needed for each sampled event, only a selection of a pretabled value.

### F. overall simulation time

The simulation should provide useful dose distribution information on a clinically realistic timescale. For our current implementation, we limit the time for simulation to 3 hours using a Mac Pro workstation (Apple Inc., Cupertino, CA) with a Quad‐Core Intel Xenon processor (Intel, Santa Clara, CA) running at a clock speed of 3.2 GHz. The four core processor has 256 KB of L2 Cache per core, and a total of 8 MB of L3 Cache. The physical memory available for computation is 24 GB. In this time period at the algorithm's current state of optimization, the number of initiated gamma events is ∼2 billion. The 3‐hour time limit was determined through an analysis of our current clinical workflow and represents a realistic time frame in which simulation could be useful in our TBI planning process. However, noise in the simulation can be greatly reduced by increasing the number of events through increasing calculation times, and the overall allowed calculation time could be adjusted on a case‐by‐case basis.

### G. Water phantom and film measurements

**Figure 2:**
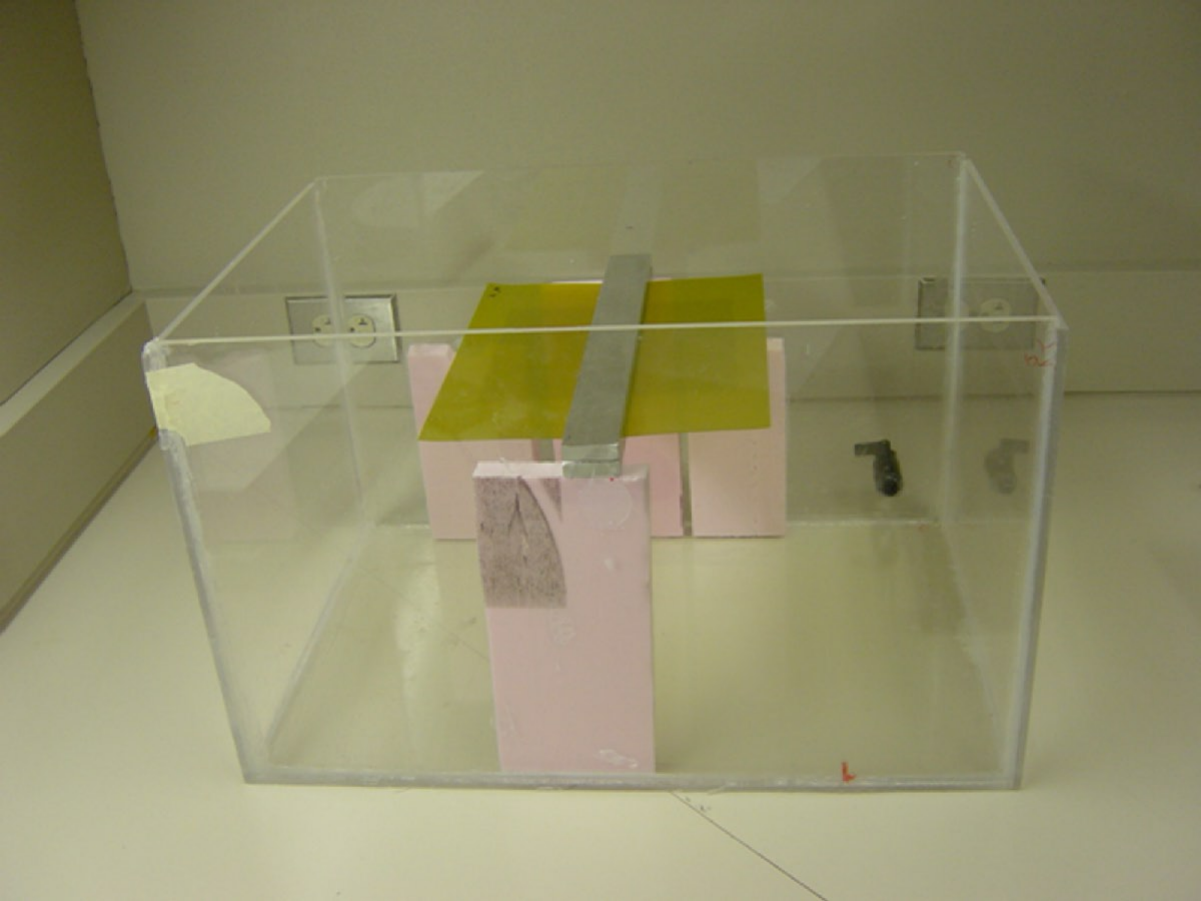
The water phantom, aluminum bars, and EBT2 film. Measurements taken using EBT2 film were compared against results of simulation.

Initial validation of the simulation was performed by percent depth dose (PDD) and depth‐dose profile measurements in a water phantom. The dimensions of the water phantom are 31.8 cm×48.4 cm×30 cm. A photo of the phantom is shown in Fig. 2. A two‐piece aluminum insert was fabricated to test the ability of the code to calculate dose near and inside a high‐density object. GAFCHROMIC EBT2 dosimetry film from ISP (Wayne, NJ) was used in the measurements.^(18)^ The choice of film was dictated by its water resistance, and energy independence from 50 keV to MeV range. Dose measurements were made by placing EBT2 film inside the water phantom at various planes, with and without the aluminum insert. The film analysis was done in RIT113 V5.2 Film Analysis software.^(19)^ The films were scanned to .TIF files using an EPSON Expression 10000XL Photo flatbed scanner (US Epson, Long Beach, CA). Small calibration films were taken at doses from 0 to 380 cGy. To eliminate as much film variation as possible, all calibration films and dosimetry films were cut from a single large sheet of GAFCHROMIC film and scanned in the same orientation with resolution of 50 dpi and 48 bit RGB color. The film measurements were compared to doses calculated by the Monte Carlo algorithm, using the RIT113 Film Analysis software.

### H. RANDO phantom and film measurements

**Figure 3:**
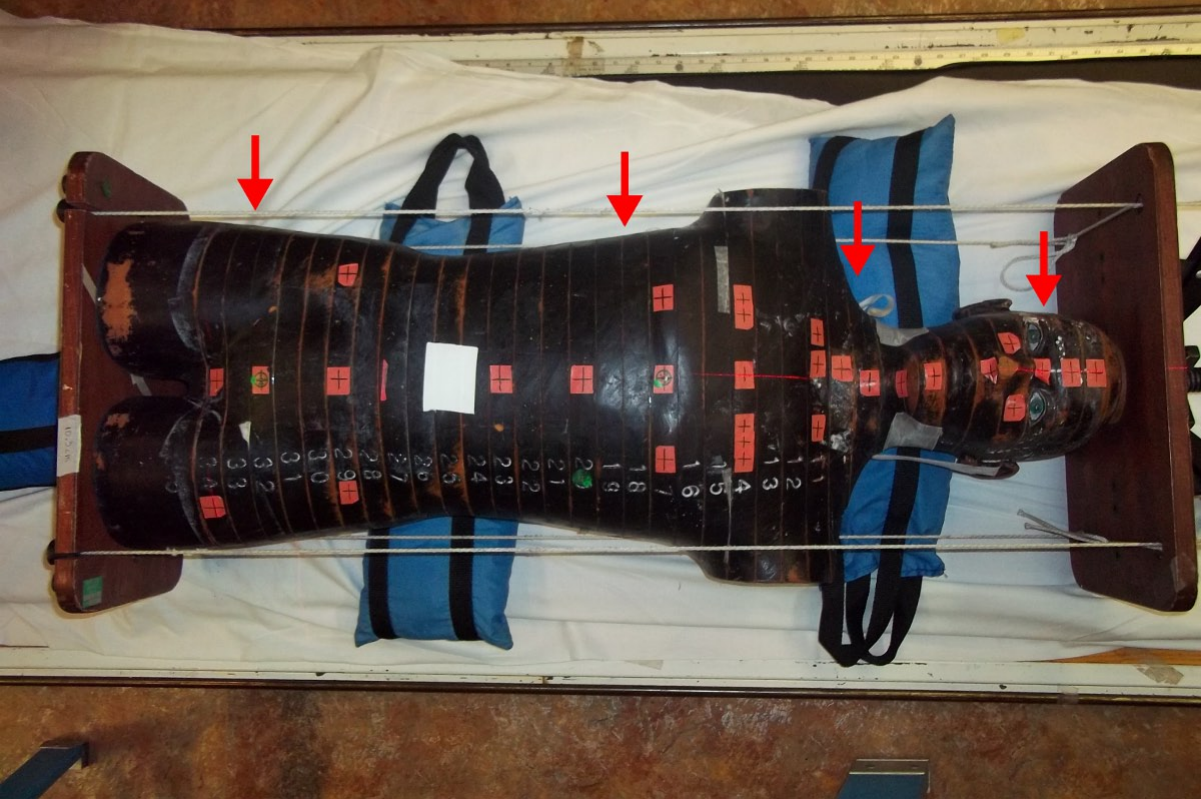
RANDO phantom on the bed and the inserted EBT2 film. Red arrows indicate film plane positions. Measurements in phantom were compared to calculated doses using the fast Monte Carlo algorithm.

Further validation of the simulation was undertaken by comparing simulation results to measured profiles in an anthropomorphic RANDO phantom (The Phantom Laboratory, Salem, NY). The RANDO phantom is comprised of 2.5 cm axial sections and is constructed of both biological and nonbiological tissue equivalent materials. This phantom was used to provide a detailed mapping of a dose distribution as similar as possible to that expected in an actual patient. For the RANDO phantom, EBT2 film was used to measure dose between the axial slices in the phantom. As shown in Fig. 3, the EBT2 film was inserted at various planes in the head, neck, lung, and pelvis of the phantom. The treatment time was 30 minutes, delivering approximately 100 cGy to the phantom at midplane. Gamma analysis was performed between the Monte Carlo simulated dose distribution and the measured dose distribution. Due to the noise limitations of the simulation, the coarse voxelization, and historical allowances for variation in TBI dose distribution (>10%), the gamma analysis is evaluated at a distance to agreement of both 3 mm and 5 mm and separately at dose deviations of 3% and 5% of maximum dose.^(10)^

### I. GEANT4 simulation and efficiency estimation

The GEANT4 simulation was performed using computer, image set, and geometry as the fast Monte Carlo simulation, including the flattening filter. The simulation was built upon the DICOM example provided with the GEANT4 framework.^(20)^ The world size was adjusted to accommodate the large treatment volume and source‐to‐surface distance. The geometry of the flattening filter was added to the world volume and the size of the voxels was adjusted to obtain the same resolution as the in‐house Monte Carlo simulation. Each voxel was represented as an independent geometrical structure with the density and material type determined from the Houndsfield units of the CT image set in the same manner as the in‐house simulation. To increase simulation speed and reduce memory overhead, the voxels are created using the G4PhantomParameterisation class. All material around the patient was designated as vacuum to prevent interactions in air as was done in the in‐house simulation. The source was constructed using an implementation of the G4GeneralParticleSource class with a dynamic assignment of the position and initial momentum of a point source to simulate the physical size of the actual ${\;}^{60}Co$ source. The physics processes included in the simulation included only G4PhotoElectricEffect and G4ComptonScattering for gammas, and G4eMultipleScattering, G4eIonisation, and G4eBremsstrahlung for electrons. The simulation was run for the same number of events as the in‐house simulation — 2 billion —and dose maps were exported to MATLAB for analysis and conversion to DICOM‐RT format readable by the RIT113V5.2 Film Analysis software.

The efficiency of both simulations, defined as $ɛ=1/(\delta^{2}T)$, where *T* is the total simulation time and $\delta^{2}$ is the variance, was estimated by recording the dose in an identical 2 cm×2 cm×2 cm box sitting approximately 5 cm deep in the abdomen region of the phantom. To estimate variance in a reasonable amount of time, ten simulations of only 20 million incident photons were run for each code, and the variance in the dose collection region was recorded (along with simulation time) to determine a rough estimate of the efficiencies of both codes.

## III. RESULTS

**Figure 4:**
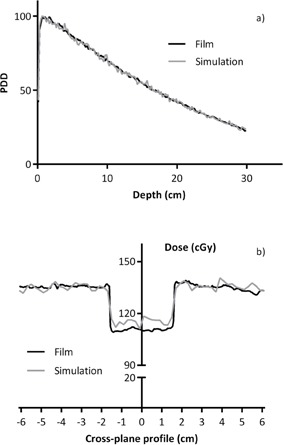
Comparison of depth dose distributions (a) between film measurement and Monte Carlo simulation. Comparison of depth dose profile (b) at depth of 3.0 cm. The middle region was between two aluminum bars.

The PDD profiles calculated using the Monte Carlo simulations in the water phantom compared favorably to those measured in the phantom. As shown in Fig. 4(a), the measured PDD along the central axis of the beam closely matches the PDD generated from the Monte Carlo code. The buildup region is obscured in the measured PDD, but the mean relative difference between the calculated and measured curve is only 3% from $D_{\text{max}}$ to a depth of 20 cm, almost entirely due to noise. When the aluminum bars with thickness of 9.5 mm are introduced into the simulation, the dose measured at the plane between two aluminum bars at a depth of 3.0 cm in the water had a mean relative difference of 4.9% on cross‐profile comparison, again thought to be mostly due to noise. This profile through the aluminum is shown in Fig. 4(b). In this profile the simulation seems to slightly broaden the interface between the water and the aluminum, which could be a result of the very simple electron transport used which ignores lateral scatter and interface effects.

**Figure 5:**
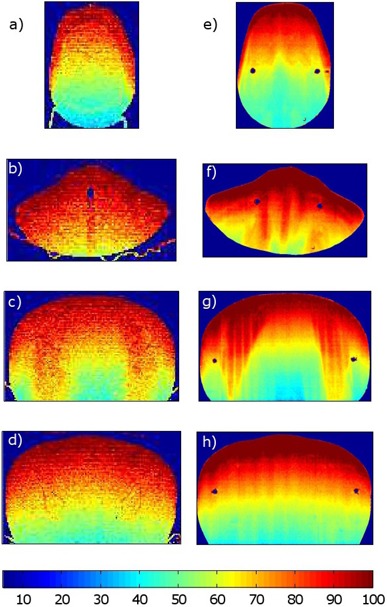
Dose distribution simulated using the Monte Carlo code in phantom head (a), neck (b), lung (c), and pelvis (d). Dose distribution measured in phantom head (e), neck (f), lung (g), and pelvis (h). Scaling factors for each film plane were independently adjusted to obtain the best gamma pass rate when comparing measured to simulated dose.

**Figure 6:**
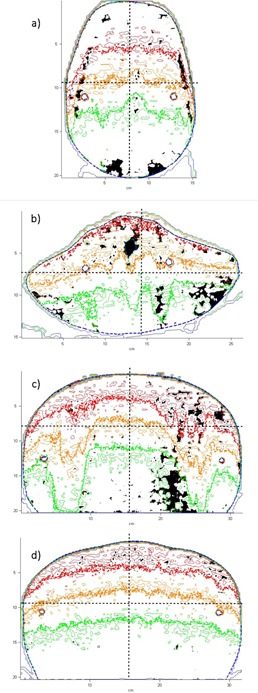
A combined isodose curve and gamma pass‐rate plot for the in‐house Monte Carlo code and film measurements in phantom for head (a), neck (b), lung (c), and pelvis (d). Dose scale is relative. Thick curves correspond to film measurements, thinner curves to the simulation. Dashed lines represent profile locations taken in each slice. Black areas indicate regions where the gamma index is >1 using the 3 mm/3% criteria.

For the RANDO phantom, measured films were scanned directly into RIT113 film analysis software. The dose distributions for the four simulated planes are displayed in Figs. 5(a) to 5(d). The corresponding measured dose planes are presented in Figs. 5(e) to 5(h). In these initial results, the raw dose values determined in the simulation are scaled such that the match in overall distributions is optimal. Qualitatively all the simulated distributions look similar to those measured. The gamma results for all planes are summarized in Table 1. Despite the low resolution of the simulation matrix, the high resolution of the film data results in the gamma pass rates varying between 91% and 99% at 3 mm, 3%. When the criteria are extended to 5 mm, 5% the pass rates are all >99%. Figures 6(a) to 6(d) present an overlay plot of the isodose distributions for both the film and the simulation. The areas in the figures where the gamma index exceeded 1 for the 3 mm, 3% criteria are colored black.

**Table 1:** Gamma pass rates for film planes in RANDO phantom.

		3%/3 mm	5%/5 mm
Film/Simulation	Head	95.0	99.5
	Neck	91.9	99.2
	Thorax	94.0	99.5
	Pelvis	99.0	99.9
Simulation/GEANT4	Head	68.8	97.2
	Neck	77.0	95.4
	Thorax	64.7	90.4
	Pelvis	78.7	98.8

The gamma comparison of the simulation results to those obtained using GEANT4 are presented in Table 1. Due to the noise and low resolution of both simulations, the gamma pass rates at 3 mm and 3% vary between 65% and 79%. When the pass rate criteria are extended to 5 mm and 5%, the pass rates vary between 90% and 99%. 7, 8 present the horizontal and vertical profiles for the film and both simulations. The position of the profiles can be seen as the black dashed line in Fig. 6. The mean relative differences between the profiles are presented in Table 2. For each slice, the mean relative difference between any of the profiles is <2%. The in‐house and GEANT4 simulations agree slightly better with each other than either does with film measurements.

The simulation time for the in‐house code was a factor of ∼30 shorter than that of the GEANT4 code for the same number of initial events. However, the variance of the in‐house code as estimated with the regional dose measurements was a factor of ~ three times larger. Thus the efficiency of the in‐house code was only ~ ten times that of the GEANT4 code despite its much shorter computational time.

**Figure 7:**
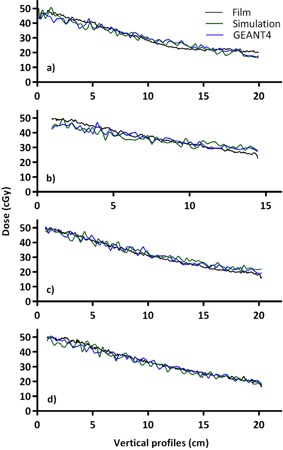
Vertical dose profiles in phantom from film, in‐house simulation, and GEANT4 in head (a), neck (b), lung (c), and pelvis (d). Doses were scaled to 50% of maximum to better display differences in curves.

**Figure 8:**
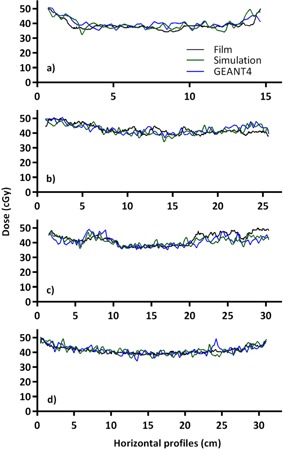
Horizontal dose profiles in phantom from film, in‐house simulation, and GEANT4 in head (a), neck (b), lung (c), and pelvis (d). Doses were scaled to 50% of maximum to better display differences in curves.

**Table 2:** Mean relative differences in the profiles taken across the measured slices.

		*Vertical (%)*	*Horizontal (%)*
Film/Simulation	Head	1.8	1.3
	Neck	1.9	1.3
	Thorax	1.8	1.4
	Pelvis	1.2	1.0
Simulation/GEANT4	Head	1.2	1.2
	Neck	1.3	1.2
	Thorax	1.3	1.2
	Pelvis	1.3	1.2
Film/GEANT4	Head	1.7	1.4
	Neck	1.4	1.3
	Thorax	1.3	1.5
	Pelvis	1.0	0.9

## IV. DISCUSSION

We believe that the comparisons between measured and simulated dose distributions illustrate that the fast Monte Carlo algorithm developed here is capable of calculating dose in heterogeneous tissue. While the simulation clearly requires further refinement, the approximations in the evaluation of the physical interactions that have been used to increase the speed of calculation appear to provide an acceptable physics framework for calculating dose for this particular TBI application.

Much work still needs to be done before any clinical implementation could be considered. Electron transport as implemented in this code leaves out many corrections applied in other codes for energy‐straggling, path length deviation, lateral scatter, and interface effects.^(21)^ In the design of the algorithm, it has been assumed that these corrections are unnecessary when considering the accuracy required for TBI dose calculations. However, this assumption may be false when considering the cumulative effects of multiple fraction treatments on the dose to important normal structures (e.g., low‐density lung tissue). A more detailed study of the specific clinical consequences that would result from inaccurate dose distribution calculation must take place before such a system could be used clinically.

The magnitude of the fluctuations in the calculated doses is directly linked to the limited number of events. This can be improved by simply increasing the number of simulated gamma rays, which is an available option only when the calculation speed has been optimized. The current calculation speeds are ∼30 times faster than an equivalent voxel‐based calculation GEANT4, while the efficiency is estimated to improve by only a factor of ∼10. Despite seemingly similar levels of noise throughout the simulations at the number of events used, the computational time decrease is only a limited success since GEANT4 is not optimized for this type of computational situation, and there are many other Monte Carlo codes much faster for voxelized dose calculations.^(22)^ The calculation speed must be further increased to provide smoother distributions within a reasonable clinical time frame. The simplest way to accelerate the algorithm is to choose larger step sizes. Increasing the step size from 1 mm to 2 mm effectively doubles the calculation speed. The voxel dimensions also play a significant role in calculation speed. For the results presented here, the voxel dimensions are 2.54 mm×2.54 mm×5 mm; however, this resolution may not be necessary for our particular application. Larger voxel dimensions of 5.08 mm×5.08 mm×5 mm could provide a suitable resolution for the determination of whole body dose.

A common step to reduce simulation time is to separate the simulation of the treatment head from that of the patient dose. A version of that approach could be used in the future. However, the reason for including it in the manner here is to provide a methodology for including patient‐specific lung blocking in the calculation. In our treatment setup lung blocks, if needed, sit only a few tens of centimeters above the patient and are thus not part of the treatment head. While further improvements of the code can include the use of a presimulated phase space for the treatment head, a simplified physical model for photon interaction with the lung blocks will still be required, and this can be built upon that presented here for the flattening filter.

While the physical processes used in the code are already sparse, further reductions in those processes should also increase calculation speed. Ignoring Rayleigh scatter within the flattening filter (and any lung blocks) may prove to provide acceptable reproductions of the physical dose distributions. Further simplifying the electron transport for low‐energy photoelectron interactions (i.e., complete local energy deposition) should decrease the computational cost by reducing sampling and increasing the ratio of sampled events that contribute most to the dose.^(5)^ More ambitious methods to improve the calculation speed could include implementing further variance reduction techniques, or the use of parallel architectures and multi‐CPU and GPU implementations of the code.^23^, ^24^, ^25^, ^26^ An accurate TBI simulation in much less than 1 hour is not an unreasonable expectation.

## V. CONCLUSIONS

In this work, a custom Monte Carlo code was developed in C to simulate dose distribution by 1.25 MeV photons in heterogeneous tissue for total body irradiation using ${\;}^{60}Co$ teletherapy unit. The algorithm is based on voxel density information from CT images of patients. Within the context of the required dose accuracy for TBI, the simulations accurately predict the PDD along the central axis and the depth‐dose profile in a water phantom. In an anthropomorphic RANDO phantom, the gamma pass rates for limits of 5 mm and 5% of maximum dose were >99% for all planes measured. At present, the fast Monte Carlo code developed here is ~ ten times more efficient than an equivalent GEANT4 simulation, and accurate to within a mean relative difference of <2% for all profiles taken across axial dose distributions produced by both codes. This has been accomplished by limiting physics processes, spatial resolution, and the resolution of lookup tables within the code to provide only the accuracy needed for TBI implementation. Further additions of variance reduction techniques to the already purpose‐built lightweight code will potentially allow this code to provide clinically useful information in planning TBI at our center.
